# Correction: Bayesian space-time SIR modeling of Covid-19 in two US states during the 2020–2021 pandemic

**DOI:** 10.1371/journal.pone.0307197

**Published:** 2024-07-10

**Authors:** Andrew B. Lawson, Joanne Kim

The Supporting information files are missing labels and captions. Please see the correct Supporting information file labels and captions here.

## Supporting information

S1 Fig Daily case counts. Model risk estimates for SC under model 5A. (TIFF)

S2 Fig Daily case counts. Model risk estimates for NJ model 5B. (TIFF)

S3 Fig 3Day averaged data. Model risk estimates for SC for model 6. (TIFF)

S4 Fig 3Day averaged data. Model risk estimates for NJ for model 6. (TIFF)

S5 Fig Mobility data: Work index. Model risk estimates for SC for model 1. (TIFF)

S6 Fig Mobility data: Work index. Model risk estimates for NJ for model 1. (TIFF)
