# Supplementary figures and images for: Bayesian space-time SIR modeling of Covid-19 in two US states during the 2020–2021 pandemic
Source: PLoS One. 2022 Dec 22;17(12):e0278515. doi: 10.1371/journal.pone.0278515 (PMC9778953; doi:10.1371/journal.pone.0278515)

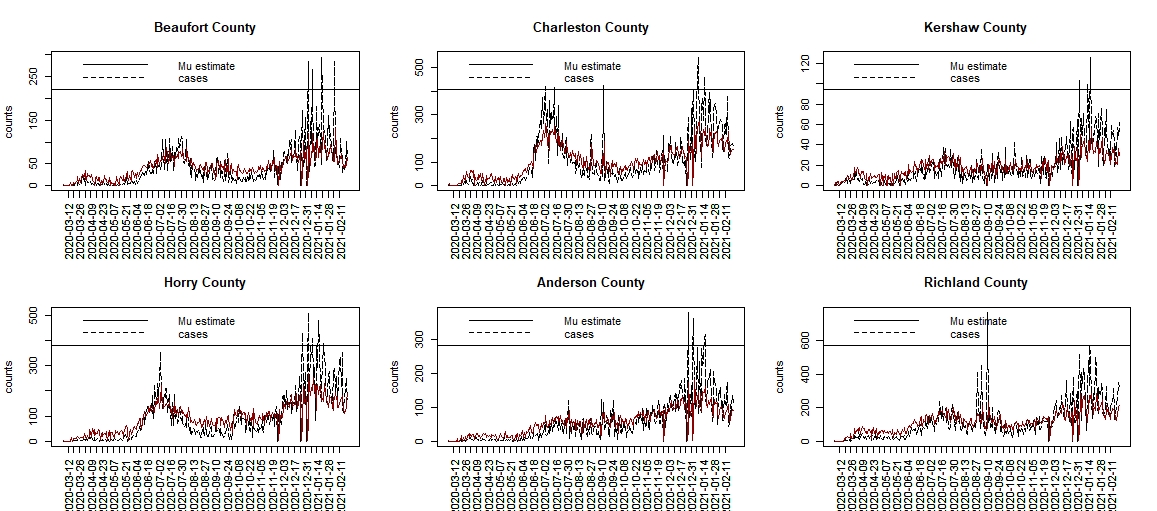

Supplement: S1 Fig — (TIFF) [file pone.0278515.s001.tiff]

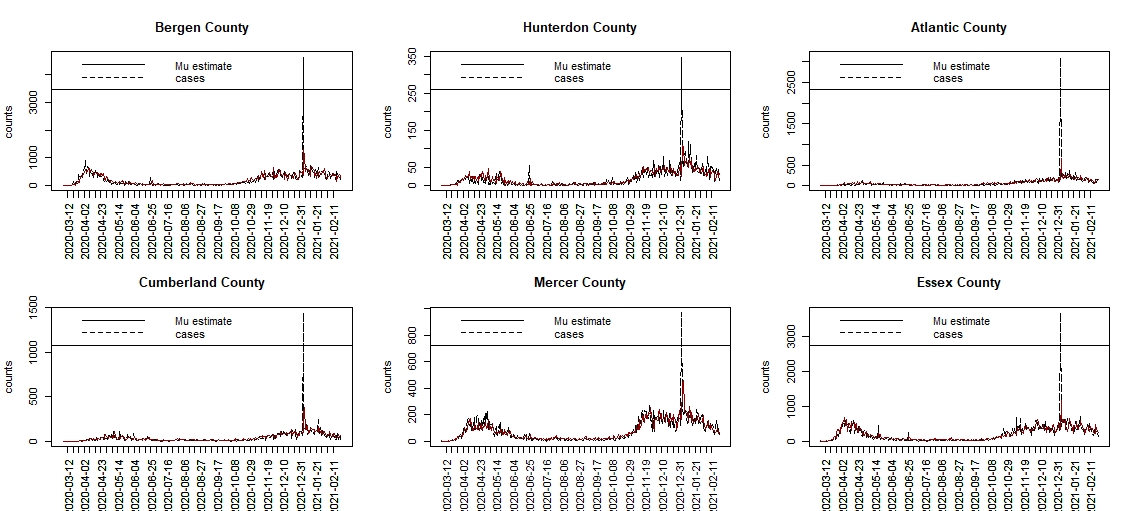

Supplement: S2 Fig — (TIFF) [file pone.0278515.s002.tiff]

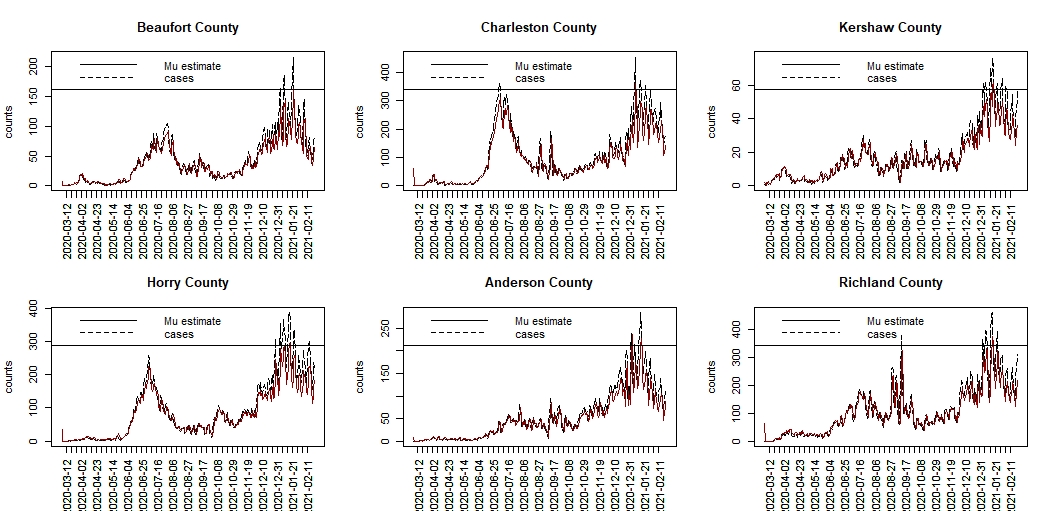

Supplement: S3 Fig — (TIFF) [file pone.0278515.s003.tiff]

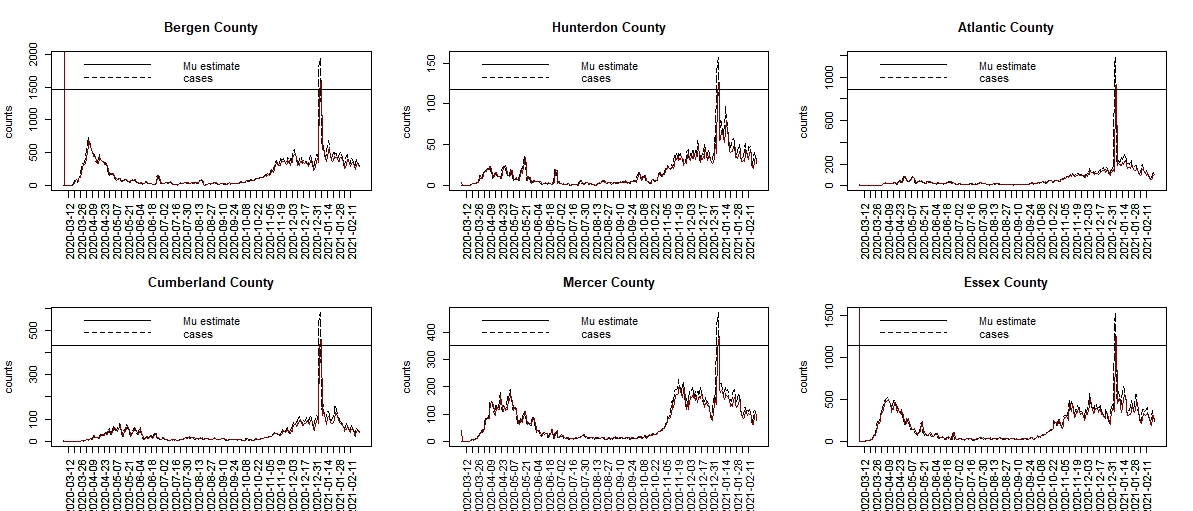

Supplement: S4 Fig — (TIFF) [file pone.0278515.s004.tiff]

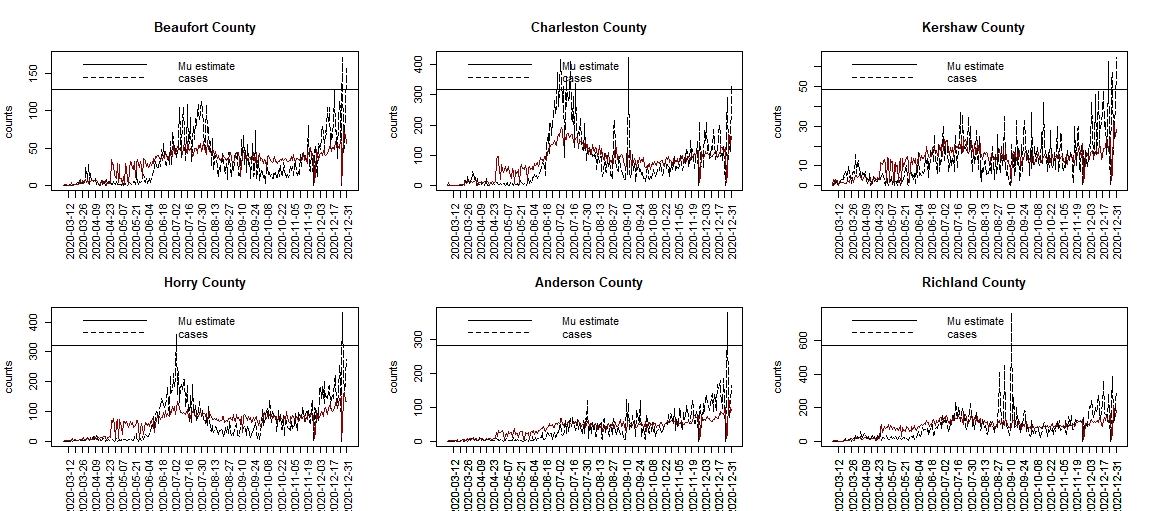

Supplement: S5 Fig — (TIFF) [file pone.0278515.s005.tiff]

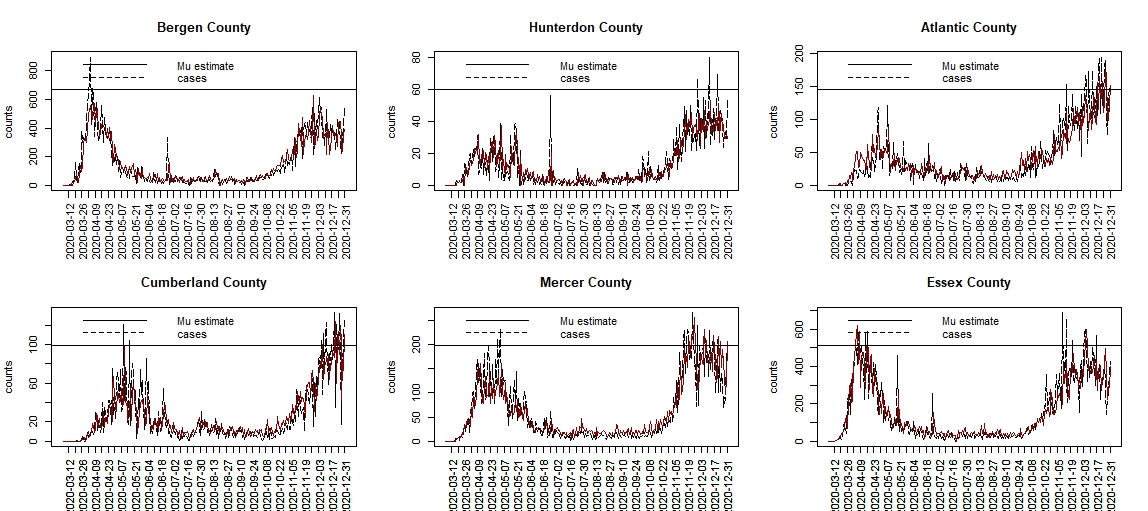

Supplement: S6 Fig — (TIFF) [file pone.0278515.s006.tiff]
